# Chronic Academic Stress Increases a Group of microRNAs in Peripheral Blood

**DOI:** 10.1371/journal.pone.0075960

**Published:** 2013-10-09

**Authors:** Manami Honda, Yuki Kuwano, Sakurako Katsuura-Kamano, Yoshiko Kamezaki, Kinuyo Fujita, Yoko Akaike, Shizuka Kano, Kensei Nishida, Kiyoshi Masuda, Kazuhito Rokutan

**Affiliations:** Departments of Stress Science, Institute of Health Biosciences, The University of Tokushima Graduate School, Tokushima, Japan; Yale University, United States of America

## Abstract

MicroRNAs (miRNAs) play key roles in regulation of cellular processes in response to changes in environment. In this study, we examined alterations in miRNA profiles in peripheral blood from 25 male medical students two months and two days before the National Examination for Medical Practitioners. Blood obtained one month after the examination were used as baseline controls. Levels of seven miRNAs (miR-16, -20b, -26b, -29a, -126, -144 and -144*) were significantly elevated during the pre-examination period in association with significant down-regulation of their target mRNAs (*WNT4*, *CCM2*, *MAK*, and *FGFR1* mRNAs) two days before the examination. State anxiety assessed two months before the examination was positively and negatively correlated with miR-16 and its target *WNT4* mRNA levels, respectively. Fold changes in miR-16 levels from two days before to one month after the examination were inversely correlated with those in *WNT4* mRNA levels over the same time points. We also confirmed the interaction between miR-16 and *WNT4* 3′UTR in HEK293T cells overexpressing FLAG-tagged *WNT4* 3′UTR and miR-16. Thus, a distinct group of miRNAs in periheral blood may participate in the integrated response to chronic academic stress in healthy young men.

## Introduction

The non-protein-coding genome is functionally important for normal development, physiology and for disease [1]. MicroRNAs (miRNAs) are a class of small non-coding RNAs approximately 22 nucleotides in length. They bind to partially complementary sites mostly within the 3′ untranslated region (UTR) of target mRNAs and suppress translation of target mRNAs or facilitate their degradation [2]. Approximately 1,000 miRNAs are expressed in human cells, and sufficiently expressed miRNAs typically target hundreds of different mRNAs. It is estimated that up to 30% of mammalian mRNA transcripts are subject to regulation by miRNAs [3]. Thus, miRNAs are one of the key regulators of eukaryotic gene expression and involved in regulation of fundamental cellular processes including proliferation, differentiation, development, and cell death [4].

Various types of stressors change the biogenesis of miRNA, activities of miRNA-protein complexes, and the expression of mRNA targets (for a review see [5]). It has also been suggested that miRNAs play an essential role in mediating stress responses [5]. Experimental animals with mutant miRNAs appear to normally develop and are viable under standard conditions, while they cannot cope with stressful conditions. This has been demonstrated in miR-14 mutant flies [6], miR-7 knockout flies [7], miR-8-inactivated zebra fishes [8], and mice deficient in miR-208 [9], suggesting that miRNAs are likely to help restore homeostasis upon sudden environmental changes.

Using a miRNA microarray, we investigated psychological stress-responsive miRNAs in whole blood from medical students taking a nationally-administered examination for academic promotion, and reported that miR-144/144* and miR-16 may participate in the regulation of systemic responses when exposed to brief naturalistic stressors in healthy young adults [10]. To confirm the reproducibility of the miRNA response in an independent stress-inducing situation, we examined changes in peripheral blood miRNA levels in medical students preparing for the National Examination for Medical Practitioners. This examination is the most stressful event for medical students, and we used this model for examining chronic stress-related changes in gene expression [11] and alternative splicing of the *glucocorticoid receptor* gene [12]. To reveal a role of miRNAs in stress responses, we also examined changes in expression of their putative target genes in leukocytes.

## Materials and Methods

### Subjects and Samples

We recruited 25 healthy male medical students (25.5±0.4 years old, mean ± SD) challenging the National Medical license examination. All experiments were conducted in accordance with the Declaration of Helsinki. The protocol and informed consent of this study were approved by the Institutional Review Board of Tokushima University Hospital, Tokushima, Japan. The experimental procedures were fully explained to each subject and written informed consent was obtained.

All subjects were in good physical health, taking no medication for at least three months prior to enrollment and during the experimental period, and had no history of psychiatric or somatic diseases. All subjects were non-smokers. Their body mass index (BMI; kilograms per meter squared) was 21.8±2.3 (mean ± SD).

Two months before, two days before, and one month after the examination, saliva was collected between 16∶00 and 17∶00 to avoid diurnal fluctuations, using Salivette sampling devices (Sarstadt Inc., Rommelsdorf, Germany) prior to the collection of blood as previously described (Kurokawa et al., 2011). Salivary cortisol levels were measured using a commercial enzyme immunoassay kit (Ciron, Tokyo, Japan) [12]. Blood was collected after saliva sampling (between 16∶00 and 17∶00) and immediately poured into PAXgene™ blood RNA tubes (Becton Dickinson, Franklin Lakes, NJ, USA). After sufficient mixing, tubes were stored for 2 h at room temperature, followed by storage at −80°C. At the same time as sample collection, all subjects answered the state version of Spielberger’s state-trait anxiety inventory (STAI) [13] for each time point. The reliability of this Japanese STAI version has been established [14].

### Measurement of miRNAs in Whole Blood

From the stored samples, total RNA containing small RNAs using the PAXgene Blood miRNA kit (Qiagen, Hilden, Germany) was extracted according to the manufacturer’s protocol. RNA concentration and purity were determined by NanoDrop ND-1000 spectrophotometer (NanoDrop Technologies, Wilmington, DE, USA). Purified RNA quality was assessed by Agilent 2100 Bioanalyzer using an RNA 6000 Nano Labchip kit (Agilent Technologies, Santa Clara, CA, USA), and RNA samples with >8.5 RNA integrity number (RIN) were used for further study. RNA samples were immediately frozen and stored at −80°C until analyzed. RNA samples were used to measure miRNA expression profiles using a human miRNA microarray (G4470C; Agilent), containing 984 miRNA probes, as previously described [10]. These data were analyzed using GeneSpring 11.5.1 (Agilent) and GraphPad Prism version 5.02 (GraphPad Software Inc., CA, USA). We selected miRNAs with fluorescence intensities >20 in at least half of the RNA samples, resulting in the detection of 160 miRNAs in the whole-blood samples. The RNA samples were also subjected to gene expression analysis using a whole human genome microarray (4×44 k, Agilent Technologies) as previously described [15]. Microarray data were analyzed by GeneSpring 11.5.1 (Agilent Technologies). The functional pathways related to the set of differentially expressed genes were assessed by Ingenuity Pathway Analysis (IPA) 9.0 (http://www.ingenuity.com) [15]. The probability of a relationship between each biological function and the identified genes was calculated by Fisher’s exact test. The level of significance was set at a *P*-value of 0.05.

### Quantitative Real-time RT-PCR (qPCR) Measurement of Target miRNAs and mRNAs

Significant changes in miRNA expression identified by microarrays were confirmed by real-time quantitative reverse transcription PCR (qPCR) using TaqMan® MicroRNA assays (Applied Biosystems, Foster City, CA, USA). The TaqMan specific miRNA retrotranscription kit (Applied Biosystems) was used to generate a specific first-strand complementary DNA from extracted total RNA (10 ng). qPCR was performed by Applied Biosystems 7500 Real-time System (Applied Biosystems). All miRNA data were normalized using *RNU48* as an endogenous quantity control. qPCR was also employed to validate the target mRNAs identified by the oligoDNA microarray. Total RNA (400 ng) from each sample was reverse-transcribed using a PrimeScript RT reagent Kit (Takara, Otsu, Japan). *WNT4*, *CCM2*, *MAK*, *FGFR1* and *SUMO2* mRNA levels were measured using SYBR Green Master Mix (Applied Biosystems). *GAPDH* mRNA was utilized as an internal control for normalization.

### YFP Reporter Plasmids

The 3′UTR of human *WNT4* was cloned into pd2EYFP-N1 (BD Biosciences, San Jose, CA). In brief, the PCR was performed using human cDNA as template. The region of the *WNT4* 3′UTR (3481–3905) containing a predicted binding site only for miR-16 (3767–3791) was amplified using the following primers (a *NotI* restriction site is underlined):

5′-AAAAAGCGGCCGCTCACCGTGGCC TGGAAATTGGCCAG-3′ (forward) and 5′-AAAAAGCGGCCGCGATAAAGAAATAATTGTTTTATCGTGC-3′ (reverse). The amplified product was subcloned into the pd2EYFP-N1 vector using a *Not1* restriction site (pd2EYFP-*WNT4* 3′UTR).

### Western Blotting

Human embryonic kidney cells (HEK293T) were cultured in Dulbecco’s modified essential medium (Invitrogen, Carlsbad, CA) supplemented with 10% fetal bovine serum. After 10 nM of control small interfering RNA (Ctrl siRNA; Qiagen, Chatsworth, CA) or precursor (pre)-miR-16 (Applied Biosystems) was transfected with Lipofectamine RNAi MAX (Invitrogen) for 24 hours, these cells were co-transfected with a pd2EYFP-N1 plasmid containing *WNT4* 3′UTR. Whole-cell lysates were prepared in a RIPA buffer (10 mM Tris-HCl, pH 7.4; 1% Nonidet P-40; 1 mM EDTA; 0.1% SDS; 150 mM NaCl) containing a protease and phosphatase inhibitor cocktail (Roche Diagnostics, Basel, Switzerland). The extracted proteins were separated by SDS-PAGE and transferred to a polyvinylidene difluoride membrane (Millipore, Billerica, MA). After blocking with 4% non-fat dry milk, membranes were incubated overnight at 4°C with anti-GFP (1∶1000; Abcam, Cambridge, UK) or anti-GAPDH (Santa Cruz Biotech., Santa Cruz, CA) antibody.

### Statistical Analysis

Data were analyzed using SPSS statistical software package version 15.0 (Chicago, IL, USA). To estimate time differences in the measures tested, repeated-measures ANOVA with Bonferroni multiple testing correction and then Tukey’s post hoc test were employed. For repeated-measures ANOVA, Greenhouse-Geisser correction was performed when violations of sphericity were detected by the Mauchly test. All significant findings remained significant when thus corrected. Therefore, we reported degrees of freedom from uncorrected analyses. Univariate correlations between identified miRNA levels and state anxiety scores, salivary cortisol levels, or mRNA levels were analyzed by Pearson or Spearman correlation coefficients. Statistical significance was set at *α* = 0.05 for all analyses.

## Results

### Changes in Anxiety and Salivary Cortisol Levels

All medical students have to pass the national medical license examination to become clinical physicians. This examination consists of a three day test and is the most stressful, important event for medical students. Samples were taken at three time points: two months and two days before the examination (pre-examination period), and one month after the examination (post-examination period). All subjects in this study were newly recruited and different from those examined in our previous studies [11], [12]. Repeated measures ANOVA showed that STAI-state scores were significantly changed during the pre- and post-examination periods (*F*(2, 48) = 22.42, *p*<0.001, *η_p_* ^2^ = 0.82). STAI-state scores measured two months (58.8±2.3; mean ± SEM, n = 25) and two days (51.3±2.3, n = 25) before the examination were fairly high, when considering the threshold value of 40 in the Japanese version of STAI [14]. Scores returned to baseline levels one month after the examination (34.6±2.3, n = 25). Tukey’s post hoc test demonstrated significantly higher state anxiety scores two months before and two days before the examination, compared with those measured one month after the examination (*p*<0.001). Repeated measures ANOVA revealed that salivary cortisol levels (*F*(2, 28) = 3.78, *p*<0.05, *η_p_* ^2^ = 0.997) also significantly responded to the examination, and the levels measured two months before (0.18±0.03 µmol/dl; mean ± SEM, n = 25), but not two days before the examination (0.16±0.03), were significantly higher than those measured one month after the examination (0.10±0.01) (*p*<0.05 by Tukey’s post hoc test). Thus, these students were likely to be under great pressure for more than two months and then were relaxed within one month after the examination. Thus, the present study seemed to be an appropriate model for the analysis of responses to chronically stressful situations.

### Changes in Peripheral Blood miRNA Levels

Among 25 male subjects, we selected 4 subjects who showed a typical stress response for microarray analysis: their STAI-state scores and salivary cortisol levels were significantly elevated during the pre-examination period. The STAI-state scores measured two months before (60.0±4.9; mean ± SEM) and two days before (50.5±6.2) were significantly higher than those measured one month after the examination (32.0±5.4), and salivary cortisol levels measured two months before (0.17±0.05 µmol/dl) and two days before (0.14±0.03) were higher than those measured one month after (0.10±0.03). Agilent human miRNA microarrays (G4470C), which have 984 miRNA probes in total, detected 160 miRNAs with fluorescence intensities >20 for at least half of the peripheral blood RNA samples. Repeated measures ANOVA without multiple testing correction revealed that levels of nine miRNAs (miR-126, miR-16, miR-1914*, miR-199a-5q, miR-20b, miR-223, miR-26a, miR-26b, and miR-29a) were significantly and time-dependently changed (Table 1). Tukey’s post hoc test revealed that all these miRNA levels measured two months and two days before the examination were significantly higher than those measured one month after the examination (*p*<0.05) (Table 1). The raw and normalized values for all samples by microarray analysis were deposited in the Gene Expression Omnibus (GEO) database (accession number: GSE49677).

**Table 1:** Examination stress-responsive miRNAs identified by miRNA array.

miRNA	2 monthsbefore exam (a)	2 daysbefore exam (b)	1 monthafter exam (c)	*p*-value1)	Time points exhibiting significant difference2)
miR-16	19044±5201	17485±3902	10704±2341	0.016	(a) vs. (c), (b) vs. (c)
miR-20b	420±117	394±91	251±79	0.043	(b) vs. (c)
miR-26a	493±77	420±78	289±46	0.011	(a) vs. (c), (b) vs. (c)
miR-26b	602±150	615±124	382±127	0.031	(a) vs. (c), (b) vs. (c)
miR-29a	98±17	89±17	57±12	0.005	(a) vs. (c), (b) vs. (c)
miR-126	724±73	284±60	164±50	0.019	(a) vs. (c), (b) vs. (c)
miR-223	10208±2116	9934±1934	7110±1289	0.017	(a) vs. (c), (b) vs. (c)
miR-199a-5p	35±6	27±4	21±3	0.004	(a) vs. (c), (b) vs. (c)
miR-1914-3p	35±8	26±9	18±4	0.024	(a) vs. (c)

Values indicate fluorescence intensities (mean ± SEM, n = 4).

1) *P*-values were calculated by repeated measures ANOVA.

2) Significantly different by Tukey’s post hoc test (*p*<0.05).

### qPCR Measurement of Identified miRNAs

**Figure 1:**
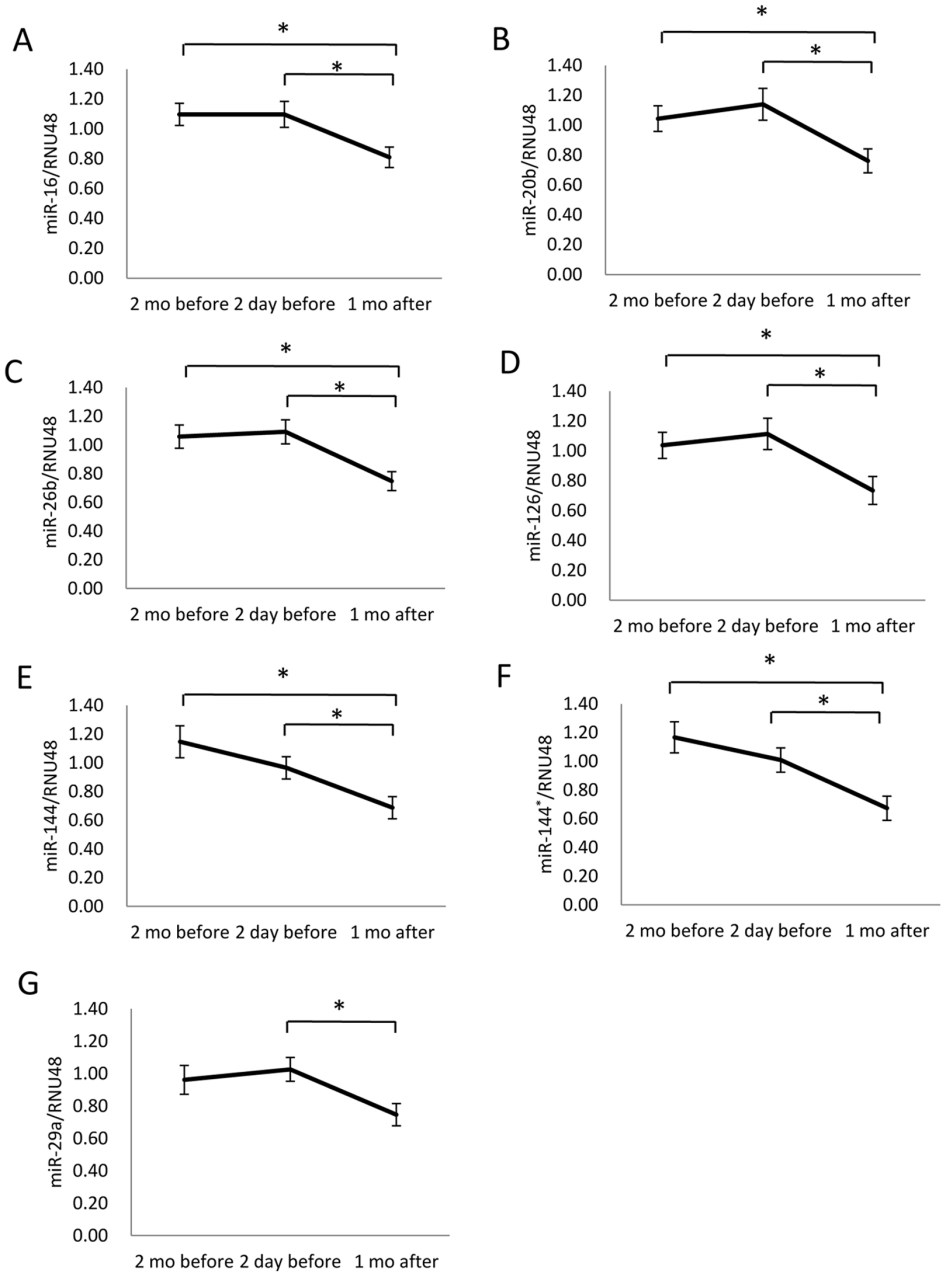
qPCR validation of time-dependent changes in seven miRNA levels. Time-dependent changes in miR-16 (A), miR-20b (B), miR-26 (C), miR-126 (D), miR-144 (E), miR-144* (F), and miR-29a (G) levels were measured by qPCR using *RNU48* as endogenous quantity control. Values are mean ± SEM (*n* = 25). *In the graphs, significantly different by repeated measured ANOVA and Bonferroni post hoc test (*p*<0.05).

Among the nine identified miRNAs, two miRNAs were not further examined, miR-1914* and miR-199a-5q, since these two miRNAs had insufficient expression levels based on their fluorescence intensities (<50) (Table 1). miR-144 and miR-144*, which had been identified as miRNAs responsive to an examination for academic promotion [10], were selected for inclusion in the further assessed miRNAs. Consequently, time-dependent changes in nine miRNA levels (miR-16, miR-20b, miR-26a, miR-26b, miR-29a, miR-126, miR-223, miR-144 and miR-144*) in 25 subjects were measured by qPCR using *RNU48* as an endogenous quantity control. Repeated measures ANOVA with multiple testing corrections revealed that miR-16 (*F*(2,48) = 7.155, *p* = 0.0019, *η_p_* ^2^ = 0.86), miR-144 (*F*(2,48) = 9.138, *p* = 0.0004, *η_p_* ^2^ = 0.91), miR-144* (*F*(2,48) = 11.80, *p*<0.0001, *η_p_* ^2^ = 0.97), miR-126 (*F*(2,48) = 6.800, *p* = 0.0025, *η_p_* ^2^ = 0.98), miR-20b (*F*(2,48) = 8.256, *p* = 0.0008, *η_p_* ^2^ = 0.90), miR-26b (*F*(2,48) = 11.61, *p* = 0.0001, *η_p_* ^2^ = 0.86) and miR-29a (*F*(2,38) = 3.880, *p* = 0.0293, *η_p_* ^2^ = 0.95) significantly responded to the examination stress, while miR-26a and miR-223 did not. As shown in Fig. 1, Bonferroni post hoc test showed that the levels of miR-16, -20b, -26b, -126, -144, and -144* measured two months and two days before the examination were significantly higher, and miR-29a levels were significantly elevated only two days before the examination, when compared with those measured one month after the examination (Fig. 1). In addition, we measured *U6 snRNA* levels as another internal quantity control. The results using *U6 snRNA* as an internal control were similar to those using *RNU48* (Figure S1). These results suggested that a prolonged stressful situation might persistently increase these specific miRNAs in peripheral blood.

### Correlation between miRNA Levels and STAI-state Scores or Salivary Cortisol Levels

**Figure 2:**
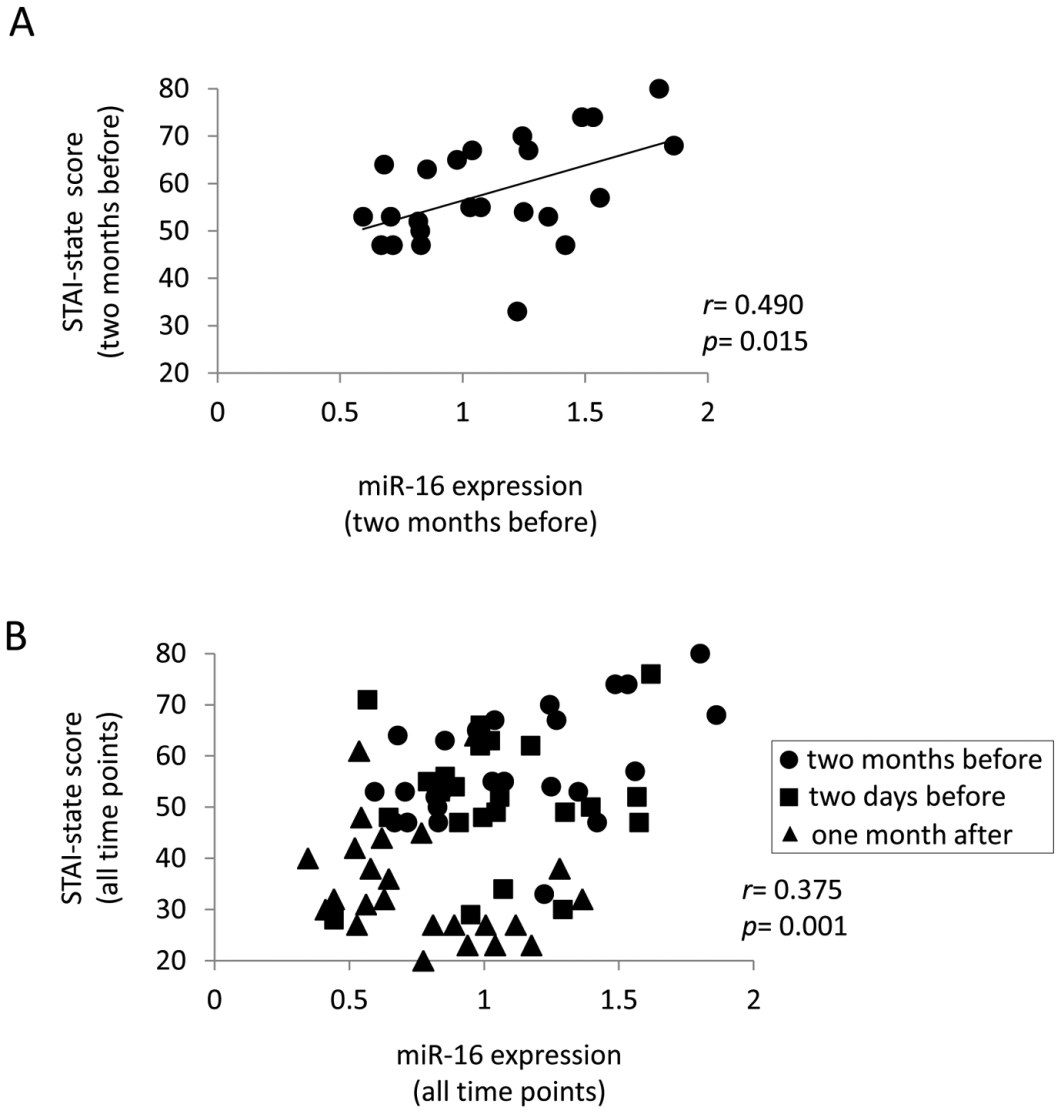
Correlation between miR-16 levels and STAI-state scores. (**A**) Correlation between miR-16 and STAI-state scores two months before the examination in 25 students was analyzed by Pearson correlation coefficients. miR-16 levels normalized by *RNU48* were significantly and positively correlated with STAI-state scores. (**B**) Correlation between miR-16 levels and the state anxiety scores measured in all three time points in 25 students was analyzed by Pearson correlation coefficients. The closed circle, square, and triangle indicate two months before, two days before, and one month after the examination, respectively.

To assess that the miRNA response constituted a part of the integrated stress response, correlations were analyzed between seven miRNA levels and STAI-state score for the individual students, and found that miR-16 levels were significantly and positively correlated with STAI-state scores two months before the examination (Fig. 2A), at which time the anxiety score showed the highest values. Moreover, as shown in Fig. 2B, miR-16 levels and the state anxiety scores measured for the individual students at all three time points were also significantly and positively correlated, suggesting that miR-16 might be a new biomarker for anxiety. In contrast, no significant correlation was observed between the other six miRNAs and the state anxiety scores at any time points. In contrast to the correlation between miRNA levels and STAI-state scores, no significant relationship was detected between the seven miRNAs and salivary cortisol levels.

### Changes in Expression of mRNAs Including miRNA Targets

To examine whether changes in the identified miRNAs were associated with altered expression of their target mRNAs in leukocytes, gene expression in peripheral blood was measured at each time point using a whole human genome microarray. Microarray analysis showed that 18,861 probes had fluorescence intensities higher than a cut-off value of 20 among all samples. Repeated measures ANOVA without multiple testing correction revealed that the expression levels of 1,356 probes were significantly and time-dependently changed (*p*<0.05). Tukey’s post hoc test showed that 1,023 mRNA levels measured two months and two days before the examination were commonly and significantly altered compared with those measured one month after the examination. Ingenuity Pathway Analysis ranked 1) Cell Death and Survival (*p* = 2.16E-07), 2) Gene Expression (*p* = 5.74E-07), and 3) Infectious Disease (*p* = 2.46E-06) as the top-three biofunctions related to the set of affected genes.

Using TargetScan (http://www.targetscan.org/), we searched for maximal local complementarity alignment between the seven miRNAs and 3′UTR sequences of putative mRNA targets, and found 1,112 predicted mRNA targets after filtering with context percentile >90.0 and their conservation. Of the 1,023 differentially expressed genes between pre- and post-examination, 58 (6%) were included in the 1,112 putative targets for the seven miRNAs. Among them, the levels of 28 mRNAs were inversely correlated to changes in the miRNA levels. Among the 28 putative targets, changes in mRNA levels of miR-16 targets (*WNT4* and *FGFR1*), a miR-20b target (*CCM2*), a miR-16 or miR-144 target (*MAK*), and miR-144 target (*SUMO2*) were validated. Repeated measures ANOVA and Bonferroni post hoc test demonstrated that preparation for the examination caused small, but significant decreases in *WNT4* (*F*(2,46) = 4.805, *p* = 0.0127, *η_p_* ^2^ = 0.76), *CCM2* (*F*(2,48) = 4.546, *p* = 0.0156, *η_p_* ^2^ = 0.94), *MAK* (*F*(2,48) = 3.801, *p* = 0.0294, *η_p_* ^2^ = 0.82), and *FGFR1* (*F*(2,42) = 4.638, *p* = 0.0151, *η_p_* ^2^ = 0.76) mRNA levels two days before the examination, compared with those measured one month after the examination (Table 2).

**Table 2:** Time-dependent changes in selected target mRNA levels.

Genesymbol	2 monthsbefore exam (a)	2 daysbefore exam (b)	1 monthafter exam (c)	Time points exhibiting significant difference1)	Putative regulator miRNA(s)
*WNT4*	1.008±0.021	0.961±0.015	1.050±0.032	(b) vs. (c)	miR-16
*CCM2*	0.935±0.036	0.886±0.038	0.952±0.034	(b) vs. (c)	miR-20b
*MAK*	0.781±0.037	0.763±0.037	0.873±0.041	(b) vs. (c)	miR-16, miR-144
*FGFR1*	0.922±0.032	0.939±0.027	1.059±0.056	(a) vs. (c)	miR-16
*SUMO2*	0.560±0.050	0.542±0.053	0.551±0.048	n.s.	miR-144

Values are mRNA levels (mean ± SEM, n = 25) standardized by *GAPDH* mRNA levels.

1) Statistical significance was determined by repeated measures ANOVA and Bonferroni post hoc test (*p*<0.05).

### Correlation between miR-16 Levels and Target mRNA Levels

**Figure 3:**
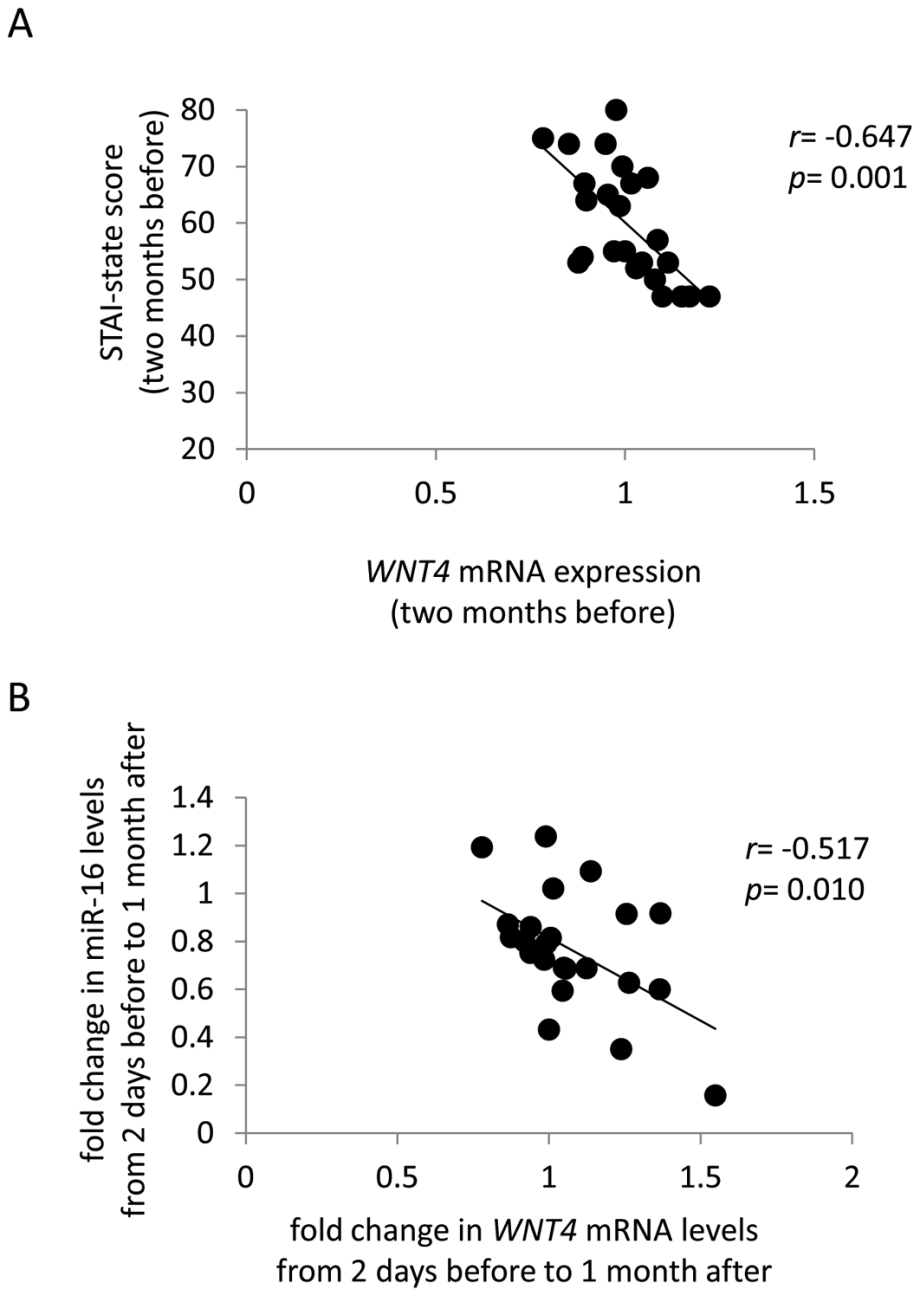
Correlation of WNT4 mRNA and miR-16 expression levels. (**A**) Correlation between *WNT4* mRNA levels and STAI-state scores two months before the examination in 25 students was analyzed by Pearson correlation coefficients. *WNT4* mRNA levels normalized by *GAPDH* mRNA levels were significantly and negatively correlated with STAI-state scores. (**B**) Correlation between fold changes in miR-16 levels from two days before to one month after the examination and fold changes in *WNT4* mRNA levels over the same time points was analyzed by Pearson correlation coefficients.

Among the seven miRNAs, miR-16 has been suggested to serve an important role in the cellular stress response [16]. Moreover, miR-16 was relatively abundant in peripheral blood and had been identified as one of the acute psychological stress-responsive miRNAs [10]. Among the miR-16 target mRNAs measured (*WNT4*, *FGFR1*, and *MAK*), *WNT4* mRNA levels were significantly and negatively correlated with STAI-state scores (Fig. 3A). Fold changes in miR-16 levels from two days before to one month after the examination were significantly (*r* = −0.517, *p* = 0.010) correlated with fold changes in *WNT4* mRNA levels over the same time points (Fig. 3B).

### WNT4 is an *in vivo* Target for miR-16

**Figure 4:**
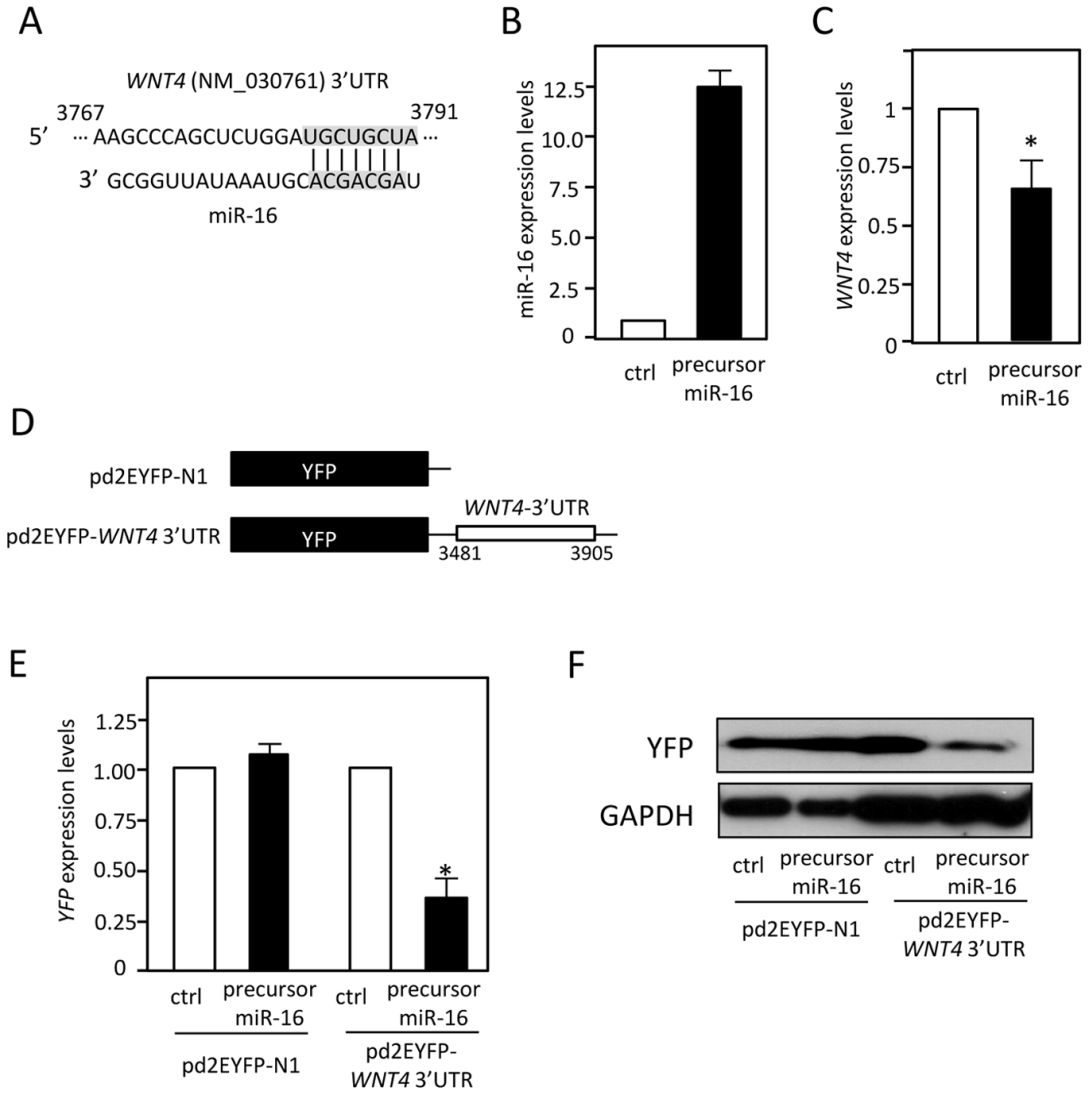
WNT4 is an *in vivo* target for miR-16. (**A**) miR-16 sequence and a predicted binding site for miR-16 in *WNT4* 3′UTR are shown. After HEK293 cells were transfected with 10 nM of control (ctrl) or precursor miR-16 for 48 h, mature miR-16 levels (**B**) and *WNT4* mRNA levels (**C**) were measured by qPCR using *RNU48* and *GAPDH* mRNA as endogenous quantity controls. Values are mean ± SD from three independent experiments. ^*^Significantly different by ANOVA and Bonferroni test (*P*<0.05) compared with those in control siRNA-treated cells. (**D**) Schematic representation of the parent reporter plasmid pd2EYFP-N1 and the pd2EYFP-*WNT4* 3′UTR reporter plasmid containing the *WNT4* 3′UTR (3481-3905). (**E**) HEK293 cells were transfected with each reporter plasmid, together with either control (ctrl) or precursor miR-16. Forty-eight hours after transfection, YFP mRNA levels were measured by qPCR using *GAPDH* mRNA as an endogenous quantity control. Values are mean ± SD from three independent experiments. ^*^Significantly different by ANOVA and Bonferroni test (*p*<0.05) compared with those in control siRNA-treated cells. (**F**) YFP and GAPDH protein levels were measured by Western blot analysis.

The 3′ UTR of *WNT4* mRNA contains a miR-16-binding site (Fig. 4A). Overexpression of precursor miR-16 reduced *WNT4* mRNA levels (Fig. 4B and C). Finally, interaction between miR-16 and 3′ UTR of *WNT4* mRNA was confirmed using HEK293 cells transfected with a plasmid encoding YFP-tagged *WNT4* 3′UTR (Fig. 4D). As shown in Fig. 4E and F, overexpression of miR-16 reduced both protein and mRNA levels of YFP-tagged *WNT4* 3′UTR, suggesting that *WNT4* is an *in vivo* target for miR-16.

## Discussion

miRNAs are involved in the control and maintenance of normal physiological functioning of the central nervous system, including brain development [17], neuron maturation [18], neurogenesis [19], [20], and synaptic plasticity [21], [22]. Both acute and chronic stressors alter miRNA expression profiles in a brain region-dependent fashion [23], [24], and changes in miRNA expression have been suggested to represent an integral part of the stress response. It is possible that specifically up-regulated miRNAs may serve as down-regulators of target mRNAs, the high expression of which may lead to detrimental effects. The stress-mediated miRNA response may activate a program of gene expression that works in concert to produce an adaptive response to the stressor. In fact, recent animal studies suggest an important role of miRNAs in stress coping reactions [5]–[9].

In a previous study, we analyzed miRNA expression profiles in peripheral blood of medical students challenged by a nationally-administered examination for academic promotion and we identified miR-144/144* and miR-16 as acute psychological stress-responsive miRNAs [10]. Presently, any psychophysiological role of these miRNAs in peripheral blood remains unclear. However, we suggested that miR-144* and miR-16 might function as down-regulators of inflammatory cytokine responses when exposed to brief naturalistic stressors [10]. Compared to the examination for academic promotion, the national examination for medical practitioners employed in this study is a much more stressful event for medical students. Most students were under pressure during the preparation period for more than six months. Thus, preparation for the national examination for medical practitioners is considered to cause chronic psychological stress [11], [12]. Even in this stress model, we could again detect up-regulation of miR-16 and miR-144/144*. Furthermore, four miRNAs (miR-20b, -26b, -29a, and -126) were newly identified as psychological stress-responsive miRNAs.

Among the identified miRNAs, the elevation of miR-16 levels is significantly and positively correlated with anxiety levels, but not with salivary cortisol levels. There was no relationship between STAI-state scores and salivary cortisol. These results suggest that the miR-16 response might be related to anxiety rather than cortisol responses. However, further studies are needed to prove this hypothesis. Recent observation indicated that miR-16 was induced by psychological stressors such as maternal deprivation and chronic unpredictable stress and reduced *brain-derived neurotoropic factor* (*BDNF*) in rat hippocampus [25]. *Serotonin transporter* (*SERT*) mRNA is a target of miR-16, and miR-16 is suggested to contribute to the therapeutic action of selective serotonin reuptake inhibitors [26]. miR-16 elevation during the pre-examination periods was associated with down-regulation of miR-16 target mRNA expression (*WNT4*, *FGFR1*, and *MAK*). *WNT4* encodes wingless-type MMTV integration site family member 4 (Wnt4) that is the first signaling molecule shown to influence the sex-determination cascade. Wnt4 also regulates neuronal differentiation and angiogenesis in the central nervous system [27]. Although any function of Wint4 in leukocytes and its psychophysiological role have not been documented, *WNT4* mRNA levels were negatively correlated with STAI-state scores two months before the examination. Moreover, *WNT4* mRNA expression did recover during the post-examination period, and this recovery was inversely correlated with the decrease of miR-16 to the baseline level over the same time points. Experiments with HEK293T cells transiently overexpressing Flag-tagged *WNT4* 3′UTR and miR-16 show that miR-16 interacted with *WNT4* 3′UTR and reduced Flag expression. Although psychophysiological role of miR16/Wnt4 in leukocytes still remains unclear, miR-16 may respond to stressful situations and participate in the stress responses during the pre-examination period.

Our subjects also had significantly increased miR-20b, miR-26b, and miR-126 levels both two months and two days before the license examination. At present, however, it is difficult to exactly explain the psychophysiological meanings of the elevation of these miRNAs. miR-20b modulates VEGF expression by targeting HIF-1α and STAT3 in MCF-7 breast cancer cells [28]. It has been reported that miR-26b expression is up-regulated in the frontal cortex of CD1 mice after acute restraint stress [24]. Recently, it has been shown that miR-29 suppresses IFN-γ production and immune responses to intracellular pathogens by directly targeting IFN-γ mRNA [29]. The elevation of miR-26b might be involved in the suppression of IFN-γ. In addition, miR-16 and miR-144^*^ might play a role in the inhibition of inflammatory cytokine response. miR-144^*^ is overexpressed in T cells from patients with pulmonary tuberculosis and is suggested to suppress IFN-γ and TNF-α production and T cell proliferation [30]. Recently, it has been shown that 3′ UTRs of *IL-6* and *TNF-α* mRNAs contain the miR-16-binding sites, and over-expression of miR-16 could significantly down-regulate TNF-α and IL-6 expression level in A549 cells [31]. In a previous study, we suggested that miR-144^*^ and miR-16 might be one of the negative regulators for inflammatory cytokine responses after exposure to naturalistic stressors [10]. In the present study, we also measured circulating IL-1β, IL-6, TNF-α, and IFN-γ at the three time points, while no significant change was detected (Table S1). Although we could not detect any significant relationship between changes in miR-20b, -26b, -29a, or -126 levels and changes in salivary cortisol levels, state anxiety, or well-known stress responsive mRNA levels, the identified miRNAs might at least in part activate a program of gene expression that works in concert to produce an integrated response to the stressor.

The fold-level changes in miRNAs in our healthy subjects are smaller, when compared with those in peripheral blood of several disorders such as Alzheimer disease [32], depression [33], and autism [34]. It is possible that disease-associated changes are likely to be larger than those observed in stress responses of healthy subjects. In addition, as described in a previous manuscript [10], individual variations of miRNA levels in peripheral blood from healthy subjects are much smaller than those of mRNA levels. We applied repeated measures ANOVA to extract candidate genes by microarray, and 78% of candidate miRNAs could be validated as significantly differentially expressed miRNAs by qPCR.

The present study is limited to a small sample size of young, healthy males and may lack statistical power. Presently, sex differences in miRNA expression profiles and their responses have not been fully documented. Our findings should be further examined using a larger number of subjects that includes both sexes. Considering potential roles of miRNAs in the regulation of gene expression under stressful conditions [10], the identified miRNAs may participate in integrated stress responses when exposed to naturalistic stressors in healthy young men. Further studies are needed to elucidate the psychophysiological meaning of the elevated miRNA levels.

## Supporting Information

Figure S1 **qPCR validation of time-dependent changes in seven miRNA levels.** Time-dependent changes in miR-16 (A), miR-20b (B), miR-26b (C), miR-126 (D), miR-144 (E), miR-144* (F), and miR-29a (G) levels were confirmed by qPCR using *U6 snRNA* as an endogenous quantity control. Values are mean ± SEM (*n* = 25). *In the graphs, significantly different by repeated measured ANOVA and Bonferroni post hoc test (*p*<0.05).
(PDF)

Table S1 Time-dependent changes in serum cytokines. Venous blood was collected after the sampling of saliva (between 16∶00 and 17∶00) and immediately poured into serumseparator tubes (Becton-Dickinson, Franklin Lakes, NJ, USA) for cytokine measurement. Separated serum was stored at −80°C until analysis. Their serum concentrations were measured using the Bio-PlexPro Human Cytokine x-plex assay (Bio-Rad, Richmond, CA, USA). Data were collected using the Bio-Plex suspension array system according to the manufacturer’s instructions (Bio-Rad) and were expressed as pg/ml.
(DOC)
