# Supplementary figures and images for: Chronic Academic Stress Increases a Group of microRNAs in Peripheral Blood
Source: PLoS One. 2013 Oct 9;8(10):e75960. doi: 10.1371/journal.pone.0075960 (PMC3794012; doi:10.1371/journal.pone.0075960)

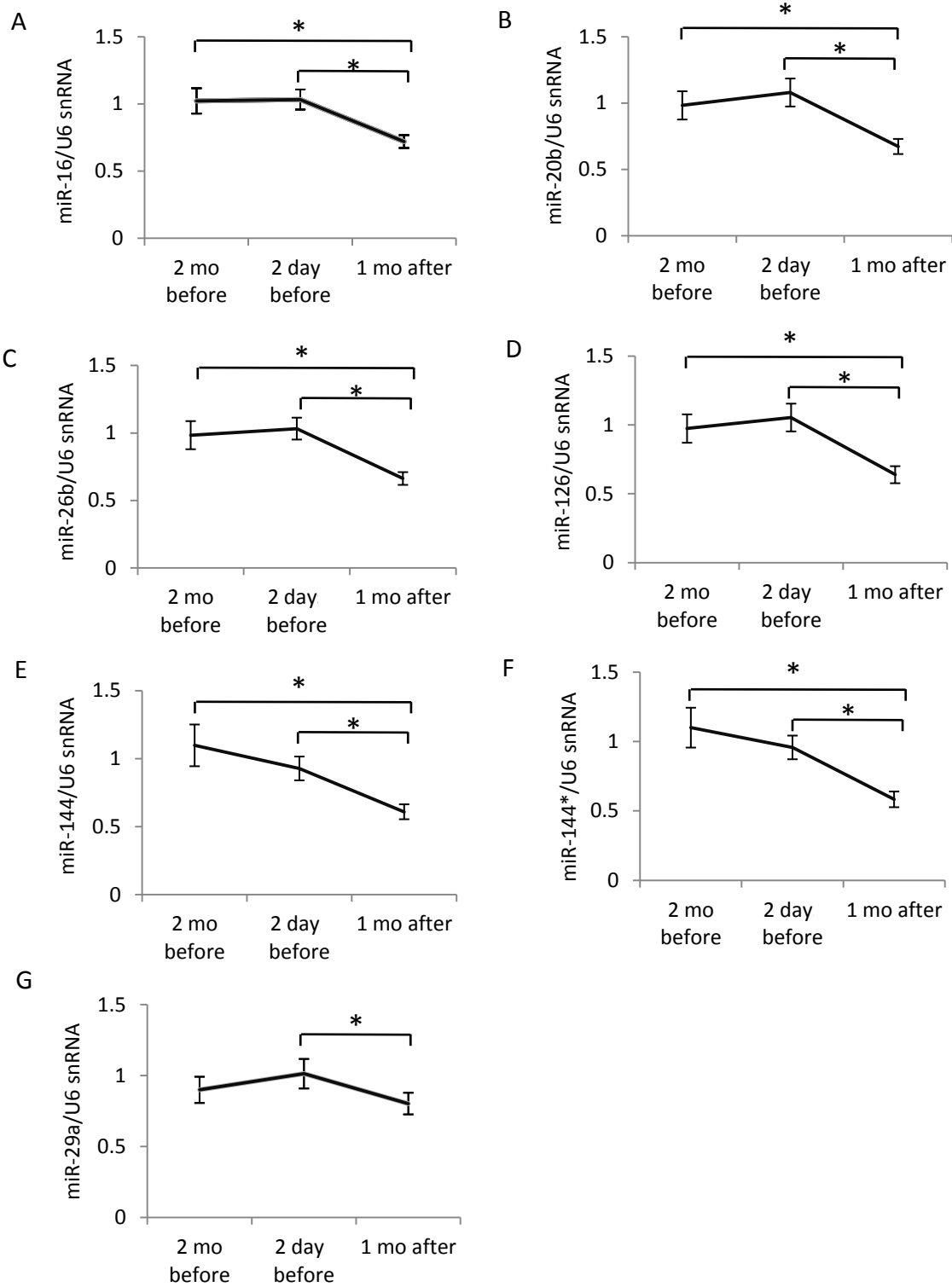

Supplement: Figure S1 — qPCR validation of time-dependent changes in seven miRNA levels. Time-dependent changes in miR-16 (A), miR-20b (B), miR-26b (C), miR-126 (D), miR-144 (E), miR-144* (F), and miR-29a (G) levels were confirmed by qPCR using U6 snRNA as an endogenous quantity control. Values are mean ± SEM (n = 25). *In the graphs, significantly different by repeated measured ANOVA and Bonferroni post hoc test (p<0.05). (PDF) [file pone.0075960.s001.pdf]
