# Supplementary material for: Chronic Academic Stress Increases a Group of microRNAs in Peripheral Blood
Source: PLoS One. 2013 Oct 9;8(10):e75960. doi: 10.1371/journal.pone.0075960 (PMC3794012; doi:10.1371/journal.pone.0075960)
Supplement: Table S1 — Time-dependent changes in serum cytokines. Venous blood was collected after the sampling of saliva (between 16∶00 and 17∶00) and immediately poured into serumseparator tubes (Becton-Dickinson, Franklin Lakes, NJ, USA) for cytokine measurement. Separated serum was stored at −80°C until analysis. Their serum concentrations were measured using the Bio-PlexPro Human Cytokine x-plex assay (Bio-Rad, Richmond, CA, USA). Data were collected using the Bio-Plex suspension array system according to the manufacturer’s instructions (Bio-Rad) and were expressed as pg/ml. (DOC) [file pone.0075960.s002.doc]

Table S1. Time-dependent changes in serum cytokines

|  |  | | 2 months before | | 2 days before | | | | 1 month after | | | | *p*-values | | | | | | | |
| --- | --- | --- | --- | --- | --- | --- | --- | --- | --- | --- | --- | --- | --- | --- | --- | --- | --- | --- | --- | --- |
|  |  | | examination | | examination | | | | examination | | | |
|  |  | |  |  | |  |  |  | |  |  |  | |  | |  | | |  |  |
| IL-1β | | 2.67 ± 0.32 (20) | | | 2.49 ± 0.33 (20) | | | | 2.39 ± 0.31 (20) | | | | p = 0.19 | | | | | | | |
|  |  | |  |  | |  |  |  | |  |  |  | | |  | |  | |  |  |
| IFN-γ | | 191.21 ± 41.89 (24) | | | 171.21 ± 41.42 (24) | | | | 192.20 ± 43.50 (24) | | | | p = 0.67 | | | | | | | |
|  |  | |  |  | |  |  |  | |  |  |  | | |  | |  | |  |  |
| TNF-α | | 92.22 ± 51.47 (16) | | | 85.63 ± 46.10 (16) | | | | 76.37 ± 35.90 (16) | | | | p = 0.44 | | | | | | | |
|  |  | |  |  | |  |  |  | |  |  |  | | |  | |  |  | |  |
| IL-6 | | 10.34 ± 3.08 (22) | | | 10.81 ± 3.63 (22) | | | | 24.05 ± 15.75 (22) | | | | p = 0.39 | | | | | | | |
| *NOTES.*  *p*-values were calculated by repeated measures ANOVA. | | | | | | | | | |  |  |  | | |  | |  | |  |  |
| Levels of serum cytokines (pg/ml) are shown as means ± SEM (number of samples). | | | | | | | | | | | | | | |  | |  | |  |  |
